# Oral Immunization of Mice with Gamma-Irradiated *Brucella neotomae* Induces Protection against Intraperitoneal and Intranasal Challenge with Virulent *B. abortus* 2308

**DOI:** 10.1371/journal.pone.0107180

**Published:** 2014-09-16

**Authors:** Neha Dabral, Martha-Moreno-Lafont, Nammalwar Sriranganathan, Ramesh Vemulapalli

**Affiliations:** 1 Department of Comparative Pathobiology, College of Veterinary Medicine, Purdue University, West Lafayette, Indiana, United States of America; 2 Departamento de Inmunología, Escuela Nacional de Ciencias Biológicas, Instituto Politécnico Nacional, Mexico City, Mexico; 3 Department of Biomedical Sciences and Pathobiology, Virginia-Maryland Regional College of Veterinary Medicine, Virginia Tech, Virginia, United States of America; East Carolina University School of Medicine, United States of America

## Abstract

*Brucella* spp. are Gram-negative, facultative intracellular coccobacilli that cause one of the most frequently encountered zoonosis worldwide. Humans naturally acquire infection through consumption of contaminated dairy and meat products and through direct exposure to aborted animal tissues and fluids. No vaccine against brucellosis is available for use in humans. In this study, we tested the ability of orally inoculated gamma-irradiated *B. neotomae* and *B. abortus* RB51 in a prime-boost immunization approach to induce antigen-specific humoral and cell mediated immunity and protection against challenge with virulent *B. abortus* 2308. Heterologous prime-boost vaccination with *B. abortus* RB51 and *B. neotomae* and homologous prime-boost vaccination of mice with *B. neotomae* led to the production of serum and mucosal antibodies specific to the smooth LPS. The elicited serum antibodies included the isotypes of IgM, IgG1, IgG2a, IgG2b and IgG3. All oral vaccination regimens induced antigen-specific CD4^+^ and CD8^+^ T cells capable of secreting IFN-γ and TNF-α. Upon intra-peritoneal challenge, mice vaccinated with *B. neotomae* showed the highest level of resistance against virulent *B. abortus* 2308 colonization in spleen and liver. Experiments with different doses of *B. neotomae* showed that all tested doses of 10^9^, 10^10^ and 10^11^ CFU-equivalent conferred significant protection against the intra-peritoneal challenge. However, a dose of 10^11^ CFU-equivalent of *B. neotomae* was required for affording protection against intranasal challenge as shown by the reduced bacterial colonization in spleens and lungs. Taken together, these results demonstrate the feasibility of using gamma-irradiated *B. neotomae* as an effective and safe oral vaccine to induce protection against respiratory and systemic infections with virulent *Brucella*.

## Introduction

*Brucella* species, Gram-negative, facultative intracellular coccobacilli, are the causative agents of brucellosis, a chronic bacterial infection in a variety of mammals, including humans. *B. melitensis*, *B. abortus* and *B. suis* are the most frequent causes of human infections [1]. These *Brucella* species are also categorized in the class B list of select agents by the CDC due to their highly infectious nature and their potential use in bio-warfare. In natural animal hosts and wildlife reservoirs, brucellosis commonly results in abortion and infertility. Chronically infected animals also shed the bacteria in milk. Human brucellosis is truly a zoonotic disease. Humans usually get infected by consuming unpasteurized contaminated milk or dairy products, or by getting exposed to infected animal tissues or secretions. Few human infections are also documented to occur through accidental exposure to live bacteria in the laboratory or inoculation of live vaccine strains used for controlling animal brucellosis in the field. Human brucellosis can manifest in a variety of clinical symptoms, starting from a subclinical infection to a protracted febrile illness which can progress to lethal endocarditis [1] and [2]. Treatment of brucellosis requires prolonged therapy with a mixture of antibiotics; even then relapses of infection are often noticed. There is no vaccine available for use in humans against brucellosis.

A safe and effective human vaccine would benefit as a prophylactic measure to protect personnel at high risk of occupational exposure to pathogenic *Brucella*, especially in *Brucella* endemic regions. Cell-mediated immunity plays a major role in enhancing the resistance against brucellosis in animals. Antigen-specific CD4^+^ and CD8^+^ T lymphocytes that secrete Th1-type cytokines such as IFN-γ and TNF-α are important in immunity against *Brucella* infection [3] [4] and [5]. In some animal species, antibodies to the O-polysaccharide (O-PS) of the lipopolysaccharide also play a role in protection against infections by *B. abortus*, *B. melitensis* and *B. suis* [6] [7] and [8]. Only live vaccines are shown to be effective in providing long term protection against brucellosis in animals. Attenuated strains such as *B. abortus* S19 and RB51, and *B. melitensis* Rev1 are used as parenteral vaccines to immunize cattle, and sheep and goats, respectively. None of the live vaccines licensed for use in domestic animals are considered safe for human application [9] and [10].

Gamma-irradiated bacteria cannot replicate but remain metabolically active, and they can be a safer alternative to live bacteria for immunization purposes. We previously showed that parenteral immunization of mice with gamma-irradiated *B. abortus* RB51 and *B. neotomae* induces protection against challenge with virulent *Brucella* spp [11] and [12]. As parenteral route of vaccination is seldom favored due to its invasive delivery and is unlikely to be a preferred route of immunization against brucellosis in humans, in this study we examined the feasibility of oral vaccination with gamma-irradiated *B. neotomae* and *B. abortus* RB51 in a prime-boost approach to induce protection against systemic and mucosal challenge infections.

We show that oral immunization with gamma-irradiated *B. neotomae* in a homologous prime-boost regimen results in production of antigen-specific antibody, cell-mediated and mucosal immune responses and increased resistance to intra-peritoneal and intranasal challenge with *B. abortus* 2308.

## Materials and Methods

### 1. Ethics statement

The protocols of the mice experiments conducted in this study were approved by the Institutional Animal Care and Use Committees at Purdue University (Approval # 1112000488) and Virginia Tech (Approval # CVM-10-048). The animal studies were carried out in strict accordance with the recommendations in the Guide for the Care and Use of Laboratory Animals of the National Institutes of Health. Blood was collected from the retro-orbital plexus from mice under anesthesia. A commercially available rodent anesthesia machine that uses oxygen and an isoflurane precision-vaporizer for supplying regulated concentration of anesthetic mixture (Vetamac, Inc., Rossville, Indiana) was used for anesthetizing mice. To reduce pain following the bleeding, a drop of proparacaine hydrochloride ophthalmic solution (Bausch & Lomb, Tampa, Florida) was placed on the eye. Mice infected with virulent *Brucella abortus* do not develop any clinical disease or show signs of suffering for the duration of the experiments conducted in this study. Therefore, no humane endpoints were utilized for the mice in this study.

### 2. Bacterial strains

*B. neotomae* strain 5K33 was obtained from the American Type Culture Collection, Manassas, VA. *B. abortus* vaccine strain RB51 and *B. abortus* virulent strain 2308 were from our culture collection. *B. neotomae* and RB51 were grown on tryptic soy broth (TSB) or tryptic soy agar (TSA) at 37°C. All experiments with *B. neotomae* were performed in a Biosafety level (BSL)-2 facility using BSL-3 practices. All experiments with virulent *B. abortus* were performed in a BSL-3 facility approved for the select agents work.

### 3. Vaccine preparation

*B. neotomae* and *B. abortus* RB51 were grown in TSB to mid log phase, and aliquots containing approximately 1×10^12^ colony forming units (CFU) ml^−1^ were exposed to 350 krads of gamma irradiation using a ^60^Co source irradiator (Gammacell 220 irradiator). The irradiated aliquots were plated on TSA to confirm the inability of the bacteria to replicate. The irradiated bacteria were stored at 4°C until use.

### 4. Immunization of mice

Groups of 4 female BALB/c mice of 4 to 6 weeks of age were used for the study. Mice were purchased from a commercial source (Harlan Laboratories, USA), and housed in cages with microisolator tops at 4 mice per cage. Feed and water were provided ad libitum. Housing conditions included standard 12 hour light/dark cycle, controlled humidity (55%) and room temperature (22°C). After 1 week of acclimatization, mice were administered with vaccine or control formulation.

The gamma-irradiated bacteria were pelleted, washed with sterile phosphate-buffered saline (PBS) and adjusted to 5×10^11^ CFU-equivalent ml^−1^. In some designated experiments, the irradiated bacteria were further diluted to concentrations of 5×10^10^ cells ml^−1^ and 5×10^9^ cells ml^−1^. A total of 200 µl of the vaccine suspension was used to vaccinate mice 15 min after oral administration of 100 µl of sterile 10% sodium bicarbonate via a disposable feeding needle attached to a 1-ml syringe.

#### 4.1. Comparison of different prime-boost immunization strategies

Mice were vaccinated by oral inoculation of 1×10^11^ CFU-equivalent of gamma-irradiated RB51 and/or gamma-irradiated *B. neotomae* in a prime-boost immunization regimen as shown in Table 1. Priming dose was administered at day 0. Booster immunizations with the same dose as the priming dose were given on days 3, 7 and day 10. A group of mice inoculated with saline was used as a negative control.

**Table 1:** Immunization schedule.

Vaccine group designation	Prime (1×10^11^ CFU)	Boost (1×10^11^ CFU)
	Day 0	Day 3, 7 and 10
IRRB51	Irradiated RB51	Irradiated RB51
IRBn	Irradiated *B. neotomae*	Irradiated *B. neotomae*
IRRB51/Bn	Irradiated RB51	Irradiated *B. neotomae*
IRBn/RB51	Irradiated *B. neotomae*	Irradiated RB51

#### 4.2. Comparison of different vaccine doses of the *B. neotomae* vaccine

Mice were vaccinated by three oral inoculations, on days 0, 3 and 10, with either 1×10^9^ CFU-equivalent or 1×10^10^ CFU-equivalent or 1×10^11^ CFU-equivalent of gamma-irradiated *B. neotomae*. A group of mice inoculated with saline was used as a negative control.

### 5. Collection of blood and intestinal secretions

Blood was collected by puncturing the retro-orbital plexus of the anesthetized mice at one and two weeks post-immunization (p.i) (1 and 2 weeks after the last booster immunization, respectively). The serum was separated and used for detection of antigen-specific antibodies by enzyme-linked immunosorbent assay (ELISA). At 2 weeks p.i., CO_2_ asphyxiation followed by cervical dislocation was used to euthanize all the mice. The small intestines were separated and the intestinal contents were collected by flushing with 3 ml of PBS and centrifuged to remove the particulate matter. The supernatants were transferred to clean tubes and were used for the analysis of antigen-specific antibody responses by ELISA. Spleens of the euthanized mice were collected aseptically and used for analysis of the antigen-specific cell-mediated immune responses by flow-cytometry.

### 6. Preparation of *B. neotomae* LPS antigen

Butanol-water extraction was used to obtain total LPS from live bacteria as previously described [13] and [14]. Briefly, 10 g of wet pellet obtained by centrifugation of live *B. neotomae* organisms was resuspended in PBS at a concentration of 0.25 g wet weight ml^−1^ followed by addition of an equal volume of water saturated butanol. The aqueous phase was collected by centrifugation at 35,000×g for 15 min. 4 volumes of cold methanol was added to precipitate the LPS. The precipitate was dissolved in 0.1 M Tris buffer (pH 8) containing 2% SDS and 2% mercaptoethanol and heated for 5 min at 100°C. Following another incubation with proteinase K for 3 hours at 60°C, cold methanol was added to precipitate LPS. The precipitate was dissolved in water. SDS-PAGE of the polyacrylamide gel was carried out to determine the purity of the LPS.

### 7. Indirect ELISA

Levels of *B. neotomae* LPS-specific immunoglobulin G (total IgG), as well as selected IgG isotypes and IgM were determined by indirect ELISA [15]. Levels of serum IgG with specificity to heat-killed RB51 total antigens was also determined. In the intestinal secretions, levels of IgG, IgM and IgA with specificity to *B. neotomae* LPS and RB51 total antigens were determined. Using carbonate buffer, pH 9.6, *B. neotomae* LPS was diluted 1 in 10, and the RB51 total antigens were diluted to obtain 1×10^9^ CFU-equivalent ml^−1^. The diluted antigens were used for coating wells (100 µl/well) of polystyrene plates (Nunc-Immunoplate with maxisorp surface). The plates were incubated at 4°C for 12 hours. They were subsequently washed with wash buffer (Tris-buffered saline (TBS), pH 7.4, with 0.05% Tween 20) and blocked with 5% skim milk in TBS. Following an incubation of 1 hour at 37°C, mouse sera with a 1∶200 dilution in blocking buffer were added to the wells (50 µl/well). All serum samples were tested in duplicates. For intestinal secretions, 1 in 10 dilution was used. The plates were incubated for 4 hours. Appropriately diluted mouse-specific horseradish peroxidase-labeled immunoglobulin conjugates (Southern Biotechnology Associates Inc, Birmingham, Alabama) were added (50 µl/well) to the wells after washing the plate four times with wash buffer. After another hour of incubation at room temperature, the plates were washed four times and 100 µl of substrate solution (TMB Microwell peroxidase substrate; KPL, Gaithersburg, MD) was added to each well. 100 µl of stop solution (0.185 M sulfuric acid) was further added to the wells in order to stop the enzymatic reaction, and the absorbance (450 nm) was recorded using a microplate reader (Molecular devices, Sunnyvale, CA).

### 8. Intracellular cytokine staining and flow cytometry analysis

Intracellular staining for IFN-γ and TNF-α was performed as previously described with some modifications [16]. Spleens were collected aseptically after euthanizing the mice and single cell suspensions of the splenocytes were prepared. ACK lysis buffer was used to lyse the RBCs and the splenocytes (10^6^ per well) were seeded in 96 well flat-bottomed plates. They were cultured with different stimulants: 10^7^ CFU-equiv. of gamma-irradiated *B. neotomae* and 10^7^ CFU-equiv. of gamma-irradiated RB51. As controls, cells were stimulated with plain medium and 2.5 µg ml^−1^ of concanavalin (ConA). They were incubated in a humidified incubator with 5% CO_2_ for 8 hours at 37°C. Subsequently, brefeldin A (Golgistop; Pharmingen) was added to the splenocytes and the plates were incubated for another 8 hours. Cells from each well were resuspended in PBS containing 1% BSA and 0.2% sodium azide (FACS buffer) and were incubated with PE-Cy7-conjugated anti-mouse CD8 antibody (BD Pharmingen, clone53-6.7) and APC-conjugated anti-mouse CD4 antibody (BD Pharmingen, clone L3T4-RM 4.5) for surface staining. They were washed three times with FACS buffer and were further stained for intracellular IFN-γ and TNF-α by incubating with PE-conjugated rat anti-mouse IFN-γ antibody and FITC-conjugated rat anti-mouse TNF-α antibody using the Cytofix/Cytoperm kit (Pharmingen). Cells stained with PE-conjugated rat IgG1 and FITC-conjugated rat IgG1 antibody served as the isotype controls. BD FACS Canto II Flow cytometer (BD Biosciences, CA, USA) was used to acquire the data which was analyzed using BD FACSDIVA version 6 software (BD Biosciences, CA, USA) and the proportion of CD4^+^and CD8^+^ T cells that secreted IFN-γ and TNF-α were determined.

### 9. Protection experiments

Protection experiments were performed at Virginia Tech in an ABSL-3 facility that was approved for work with select agents. Female BALB/c mice of 4–6 weeks of age (Harlan Laboratories, USA) were used for the studies. Mice were housed in individually ventilated cages with high-efficiency particulate arresting-filtered air. Feed and water were provided ad libitum. Housing conditions included standard 12 hour light/dark cycle, controlled humidity (55%) and room temperature (22°C). After 1 week of acclimatization, mice were administered with vaccine or control formulation.

#### 9.1. Analysis of protective efficacy of the different prime-boost immunization regimens

Groups of 5 mice were vaccinated by oral inoculation of 1×10 CFU-equiv. of gamma-irradiated RB51 and/or gamma-irradiated *B. neotomae* in a prime-boost immunization regimen; immunizations were performed as described above in section 2.4.1. A group (n = 5) of mice inoculated with saline alone served as control. Two weeks p.i, each mouse was challenged by i.p. inoculation with 1×10^4^ CFU of *B. abortus* 2308. Two weeks post challenge, the mice were euthanized and the number of bacterial CFUs in their spleens, lungs and livers were enumerated as previously described [17] and [18]. Briefly, two weeks post challenge, the mice were euthanized and their spleens, lungs and livers were collected aseptically. Organs were suspended in 1 ml of sterile PBS and were separately homogenized using tissue grinders. Ten-fold serial dilutions of the tissue homogenates were prepared in saline, and 50 µl of each dilution was plated on TSA. After 3–5 days of incubation at 37°C, the *Brucella* CFUs were counted and the bacterial burden per organ was calculated.

#### 9.2. Analysis of the protective efficacy of different vaccine doses of *B. neotomae*

Groups of 10 mice were vaccinated by three oral inoculations on days 0, 3 and 10 with either 1×10^9^ CFU-equiv. or 1×10^10^ CFU-equiv. or 1×10^11^ CFU-equiv. of gamma-irradiated *B. neotomae* (as per the Materials and Methods 2.4.2.). A group (n = 10) of mice inoculated with saline alone served as control. Two weeks p.i. (2 weeks after the inoculation of the last booster dose), five mice from each group were challenged by i.p. inoculation with 5×10^4^ CFU of *B. abortus* 2308, and the remaining five mice were challenged by i.n. inoculation with 4×10^6^ CFU of *B. abortus* 2308. Two weeks post challenge, the mice were euthanized and the bacterial loads in their spleens, lungs and livers were enumerated as described in section 2.9.1.

### 10. Statistical analyses

Absorbance values of ELISA and flow cytometry data were analyzed for differences among different vaccinated groups by performing analysis of variance with post hoc Tukey for pair-wise comparison using SPSS version 21.0 (SPSS Inc., an IBM company, USA). Following bacterial challenge experiments, student t-test modified for unequal variances between groups was carried out to compare the log transformed CFU values in organs obtained from each vaccinated group of mice with the respective saline group. *P* values of <0.05 were considered significant.

## Results

### 1. Induction of antigen-specific antibody immune responses following oral prime-boost immunization with gamma-irradiated *B. neotomae* and/or gamma-irradiated *B. abortus* RB51

**Figure 1:**
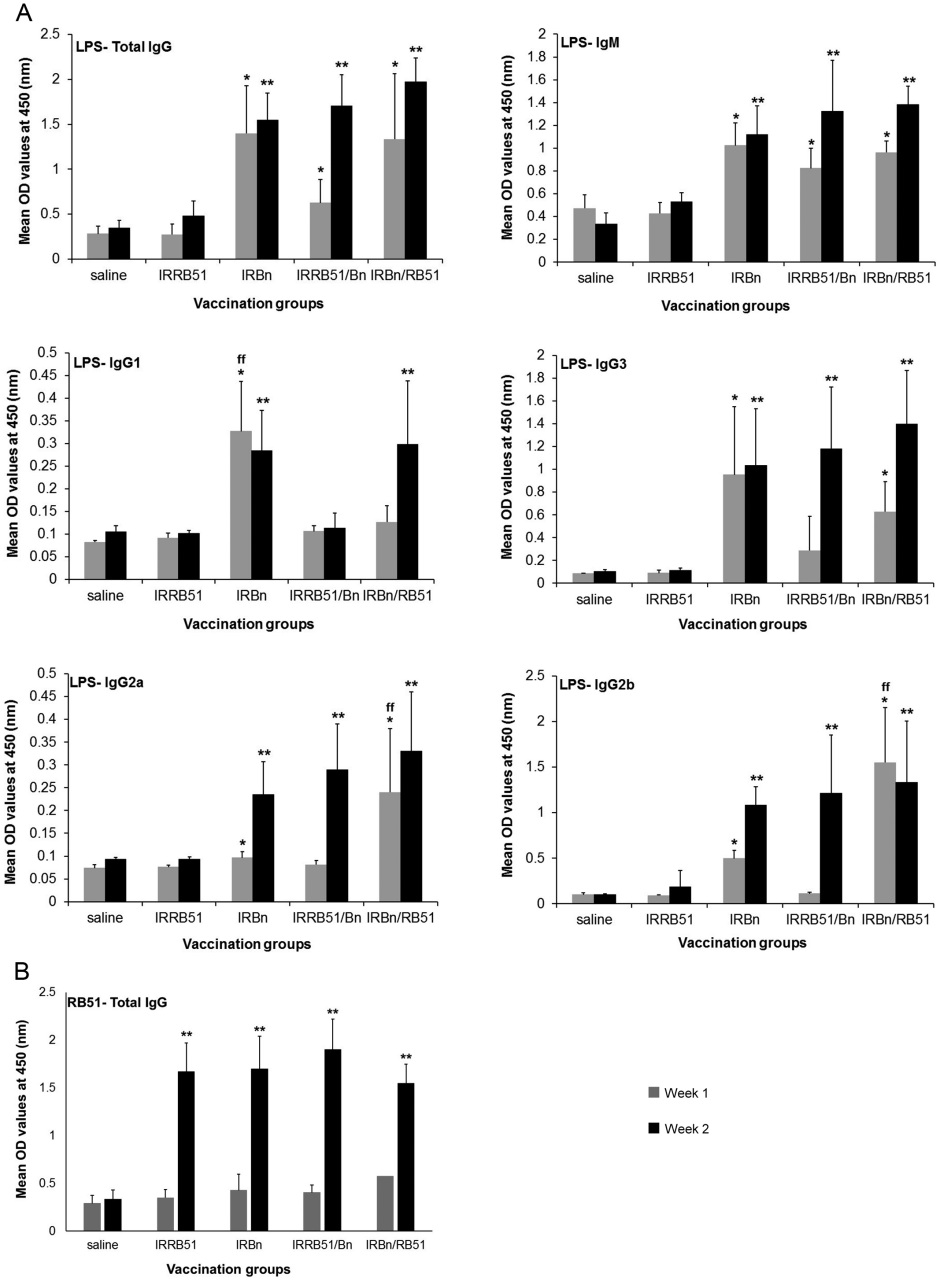
ELISA detection of IgG, IgM, IgG1, IgG3, IgG2a and IgG2b antibodies specific to *B. neotomae* LPS (A), and IgG antibody specific to RB51 total antigens (B) in serum of mice vaccinated with gamma-irradiated *RB51*, *B. neotomae*, RB51/*B. neotomae* and *B. neotomae*/RB51, or inoculated with saline. Serum samples were collected at 1 and 2 weeks after the last booster vaccination, were diluted 1 in 200 and assayed for the presence of LPS-specific (A) and RB51-specific (B) antibodies. Results are shown as mean ± standard deviation (*n* = 4) of absorbance at 450 nm of the color developed. *Significantly different from the corresponding saline group at week 1 (*P*<0.05). **Significantly different from the corresponding saline group at week 2 (*P*<0.05). ^ff^ Significantly different from the corresponding vaccination groups at week 1 (*P*<0.05).

Levels of antibodies specific to *B. neotomae*-smooth LPS and RB51 total antigens in serum samples collected at 1 and 2 weeks p.i. (1 and 2 weeks after the inoculation of the last booster dose, respectively) were determined by indirect ELISA. Immunization of mice with IRBn, IRBn/RB51 and IRRB51/Bn resulted in the induction of significantly higher levels of serum IgG and IgM antibodies specific to LPS when compared with the saline inoculated mice and the group of mice vaccinated with IRRB51 (Fig. 1A). However, there was no significant differences among the three vaccination groups (Fig. 1A). Significantly higher levels of LPS-specific IgG2a, IgG2b and IgG3 were present in the serum samples of mice immunized with IRBn and IRBn/RB51 at 1 week and 2 week p.i. (Fig. 1A). Also, mice vaccinated with IRBn and IRBn/RB51 had higher levels of LPS-specific IgG1 antibodies at 2 weeks p.i.; however, only mice vaccinated with IRBn had significantly increased levels of serum IgG1 antibodies specific to LPS at 1 week p.i. (Fig. 1A). In contrast, vaccination of mice with IRRB51/Bn did not lead to the induction of significant levels of LPS-specific IgG1 antibodies (Fig. 1A). Furthermore, significantly higher levels of LPS-specific IgG2a, IgG2b and IgG3 antibodies were detected in the serum of mice vaccinated with IRRB51/Bn only at 2-weeks p.i. (Fig. 1A).

**Figure 2:**
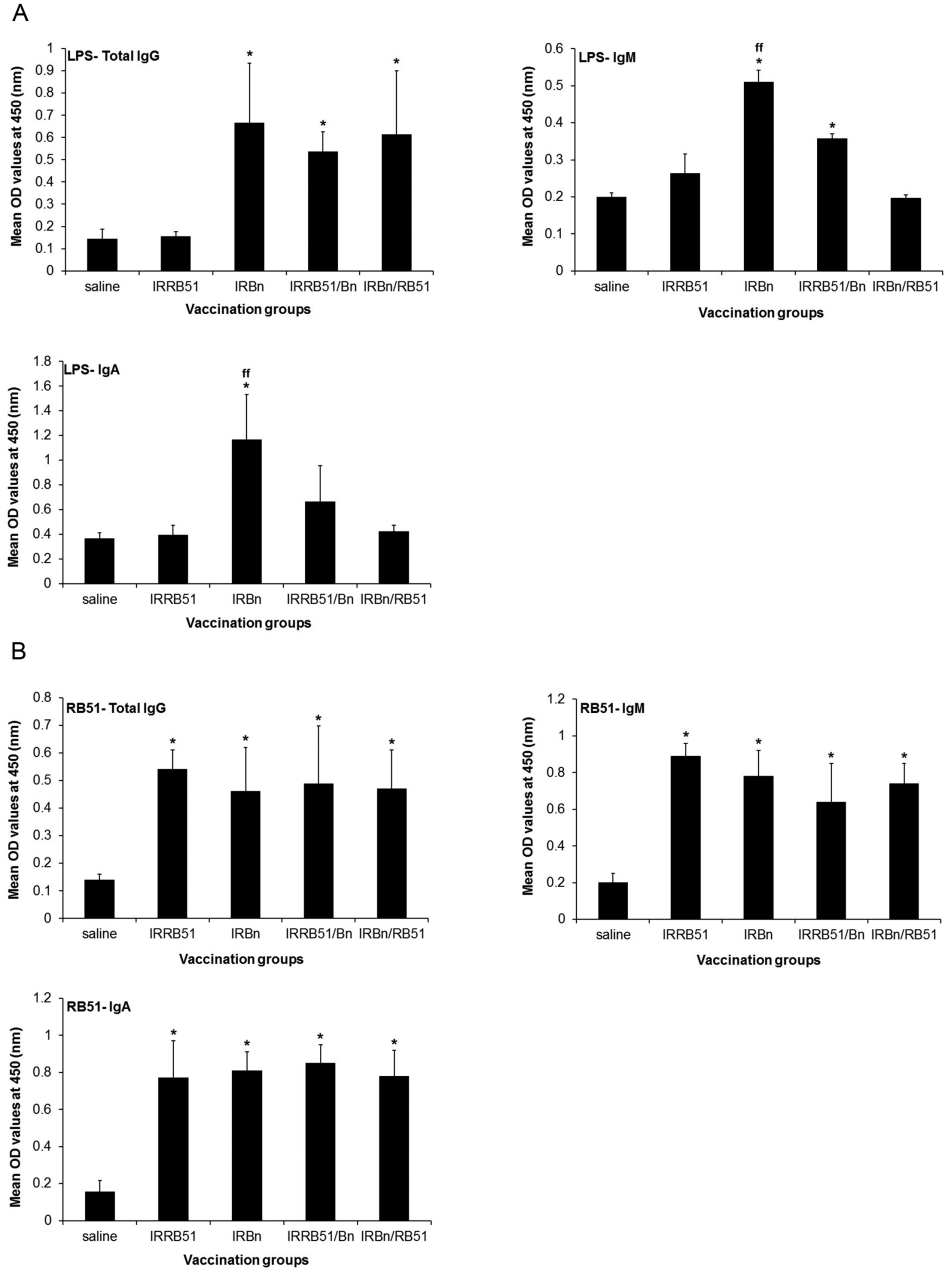
ELISA detection of IgG, IgM, IgA antibodies specific to *B. neotomae* LPS (A), and RB51 total antigens (B) in the intestinal secretions of mice vaccinated with gamma-irradiated RB51, ***B. neotomae***, RB51/*B. neotomae* and ***B. neotomae***/RB51, or inoculated with saline. Intestinal secretions were collected at 2 weeks after the last booster vaccination, were diluted 1 in 10 and assayed for the presence of LPS-specific (A) and RB51-specific (B) antibodies. Results are shown as mean ± standard deviation (*n* = 4) of absorbance at 450 nm of the color developed. *Significantly different from the corresponding saline group (*P*<0.05). ^ff^ Significantly different from the corresponding vaccination groups (*P*<0.05).

Oral immunization of mice with IRBn, IRRB51/Bn and IRBn/RB51 led to the induction of significantly higher levels of LPS-specific IgG antibodies in the intestinal secretions when compared with the saline inoculated control and the IRRB51 vaccinated group (Fig. 2A). Only vaccination of mice with IRBn resulted in the induction of significantly increased levels of LPS-specific IgM and IgA antibodies in the intestinal secretions when compared to the other vaccinated groups (Fig. 2A).

All four vaccinated groups of mice had significantly higher levels of IgG serum antibodies specific to RB51 total antigens (Fig. 1B), as well as significantly higher levels of RB51-specific IgG, IgM and IgA antibodies in the intestinal secretions (Fig. 2B) than the saline inoculated control mice. There was no difference in the levels of RB51-specific antibodies among the different groups (Fig. 1B and 2B).

### 2. Induction of antigen-specific cell-mediated immune responses following oral prime-boost immunization with gamma-irradiated *B. neotomae* and/or gamma-irradiated *B. abortus* RB51

**Figure 3:**
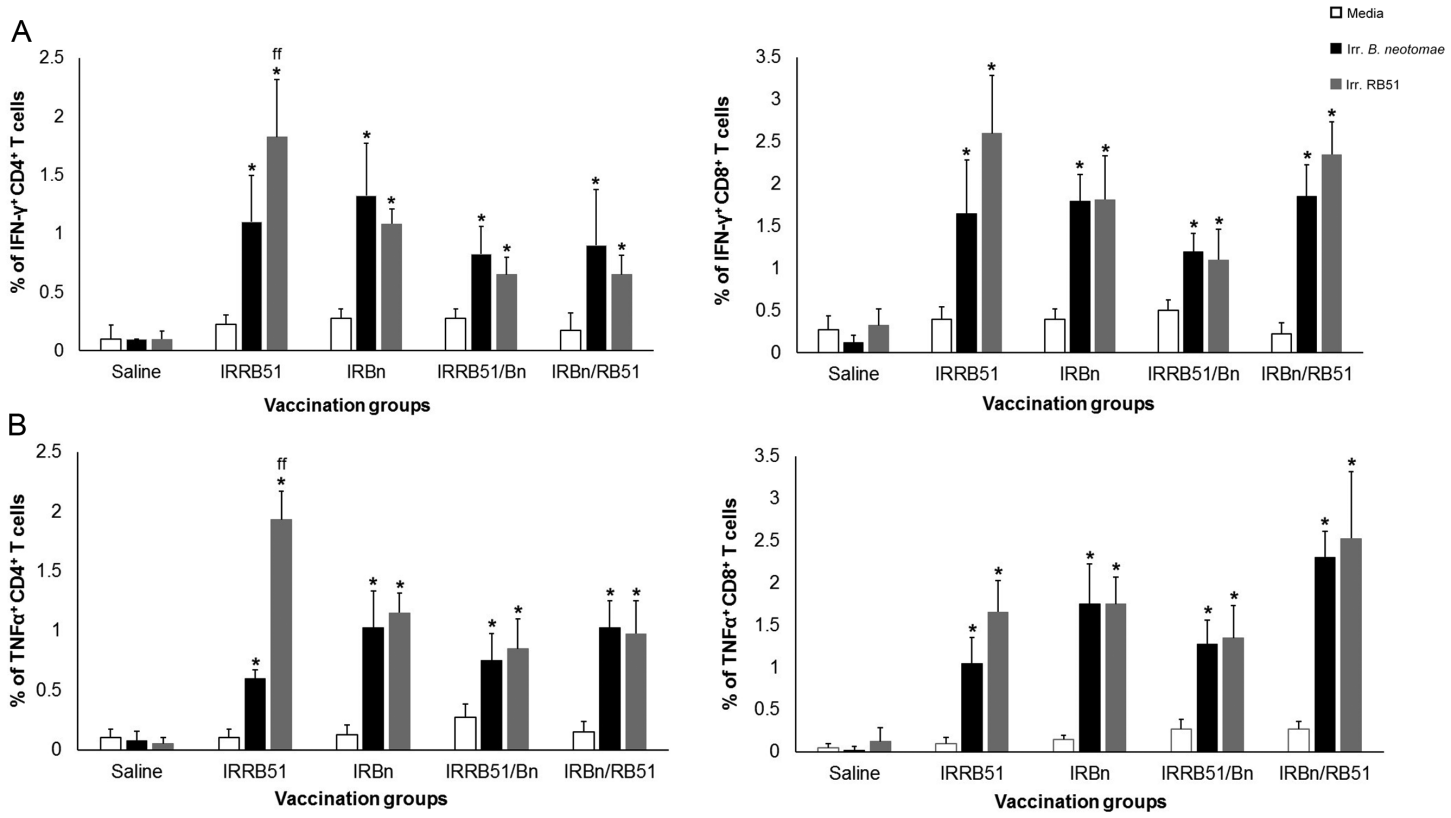
Flow cytometric analysis showing the percentage of interferon-γ secreting (A), and tumor necrosis factor-α secreting (B) CD4^+^ and CD8^+^ T cells in the spleens of BALB/c mice immunized with gamma-irradiated RB51, *B. neotomae*, RB51/*B. neotomae* and *B. neotomae*/RB51, or inoculated with saline following in vitro stimulation with media (unstimulated), gamma-irradiated *B. neotomae* and gamma-irradiated RB51. *Significantly different from the corresponding unstimulated control (*P*<0.05). ^ff^ Significantly different from the corresponding vaccination groups with irradiated RB51 stimulation (*P*<0.05).

Significantly higher proportions of antigen-specific IFN-γ-and TNF-α-secreting CD4^+^ and CD8^+^ T cells were detected in all vaccinated groups when compared to the respective unstimulated controls and the saline inoculated control group (Fig. 3A and 3B). Stimulation of the splenocytes with irradiated RB51 resulted in the induction of significantly increased proportions of RB51-specific CD4^+^ T cells secreting IFN-γ and TNF-α in mice immunized with IRRB51 than the other vaccinated groups (Fig. 3A and 3B). There was no significant difference in the proportion of IFN-γ and TNF-α secreting CD8^+^ T cells specific to RB51 and those that were specific to *B. neotomae* among the different vaccinated groups (Fig. 3A and 3B).

### 3. Protective efficacy against intra-peritoneal challenge with virulent *B. abortus* 2308 following oral prime-boost immunization with gamma-irradiated *B. neotomae* and/or gamma-irradiated *B. abortus* RB51

Immunization with IRBn and IRBn/RB51 resulted in a significant reduction in the number of bacterial CFUs of the virulent *Brucella* in the spleen after i.p. challenge with virulent *B. abortus 2308* (Table. 2). In contrast, the bacterial burden in the spleen of mice immunized with IRRB51 was not significantly different from that of the saline inoculated control group (Table. 2). All three vaccinations resulted in reduced bacterial load of virulent strain in livers (Table. 2). The bacterial loads in lungs of the vaccinated mice were not statistically different from that of the saline group (Table. 2).

### 4. Induction of antigen-specific antibody immune responses following oral homologous prime-boost immunization with different vaccine doses of gamma-irradiated *B. neotomae*

**Figure 4:**
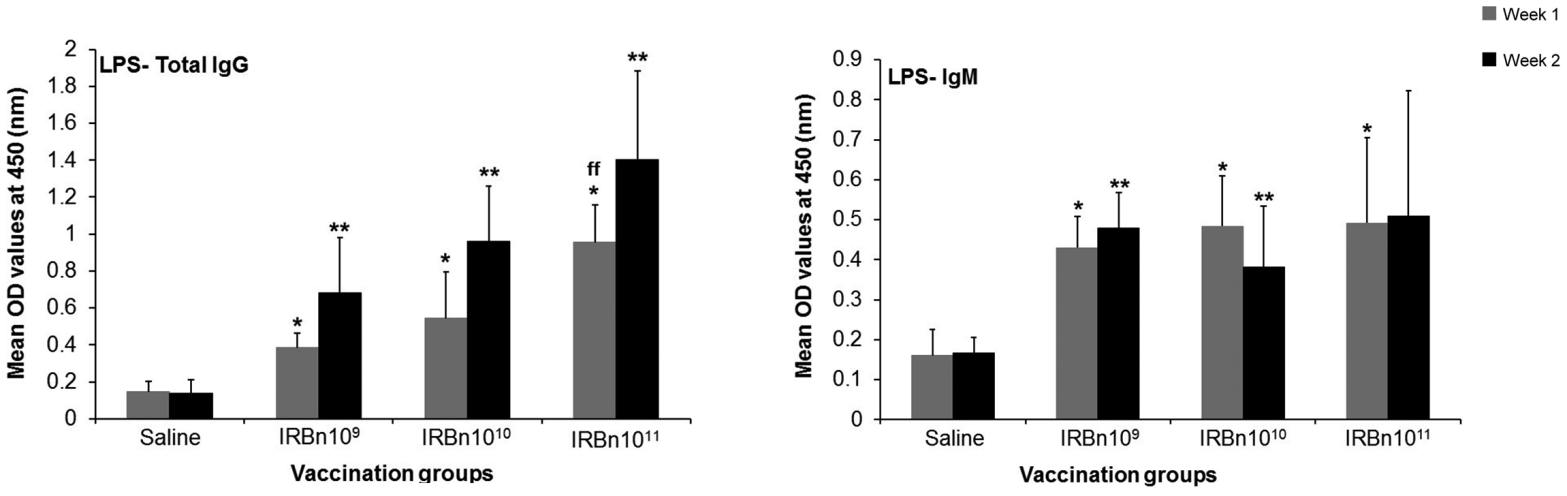
ELISA detection of IgG and IgM antibodies specific to ***B. neotomae*** LPS in serum of mice vaccinated with 10^9^ CFU-equivalent or 10^10^ CFU-equivalent or 10^11^ CFU-equivalent of gamma-irradiated *B. neotomae*, or inoculated with saline. Serum samples were collected at 1 and 2 weeks after the last booster vaccination, were diluted 1 in 200 and assayed for the presence of LPS-specific antibodies. Results are shown as mean ± standard deviation (*n* = 4) of absorbance at 450 nm of the color developed. *Significantly different from the corresponding saline group at week 1 (*P*<0.05). **Significantly different from the corresponding saline group at week 2 (*P*<0.05). ^ff^ Significantly different from the corresponding vaccination groups at week 1 (*P*<0.05).

We examined the effect of different doses of gamma-irradiated *B. neotomae* vaccine on induction of specific immune responses and protection against parenteral and mucosal challenge with virulent *B. abortus*. Three different doses, 1×10^9^ CFU-equiv. or 1×10^10^ CFU-equiv. or 1×10^11^ CFU-equiv. of gamma-irradiated *B. neotomae* were tested in a prime-boost immunization strategy, consisting of a priming dose and two booster doses. Significantly increased levels of LPS-specific IgG antibodies at 1 and 2 weeks p.i. and LPS-specific IgM antibodies at 1 week p.i. were detected in the serum of the three vaccinated groups when compared to the saline inoculated control group (Fig. 4). In addition, mice vaccinated with the 10^11^ CFU-equiv. dose developed significantly increased levels of LPS-specific IgG antibodies in the serum at 1 week p.i. when compared to the other vaccinated mice (Fig. 4).

**Figure 5:**
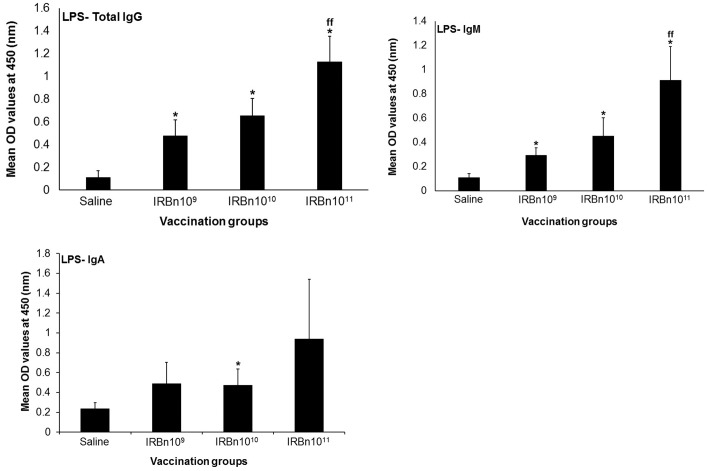
ELISA detection of IgG, IgM and IgA antibodies specific to *B. neotomae* LPS in the intestinal secretions of mice vaccinated with 10^9^ CFU-equivalent or 10^10^ CFU-equivalent or 10^11^ CFU-equivalent of gamma-irradiated *B. neotomae*, or inoculated with saline. Intestinal secretions were collected at 2 weeks after the last booster vaccination, were diluted 1 in 10 and assayed for the presence of LPS-specific antibodies. Results are shown as mean ± standard deviation (*n* = 4) of absorbance at 450 nm of the color developed. *Significantly different from the corresponding saline group (*P*<0.05). ^ff^ Significantly different from the corresponding vaccination groups (*P*<0.05).

Significantly higher levels of LPS-specific IgG and IgM antibodies in the intestinal secretions were present in all vaccinated mice (Fig. 5). Mice vaccinated with the 10^11^ CFU-equiv. dose contained the highest levels of IgG, IgM and IgA antibodies (Fig. 5).

### 5. Induction of antigen-specific CMI following oral homologous prime-boost immunization with different vaccine doses of gamma-irradiated *B. neotomae*

**Figure 6:**
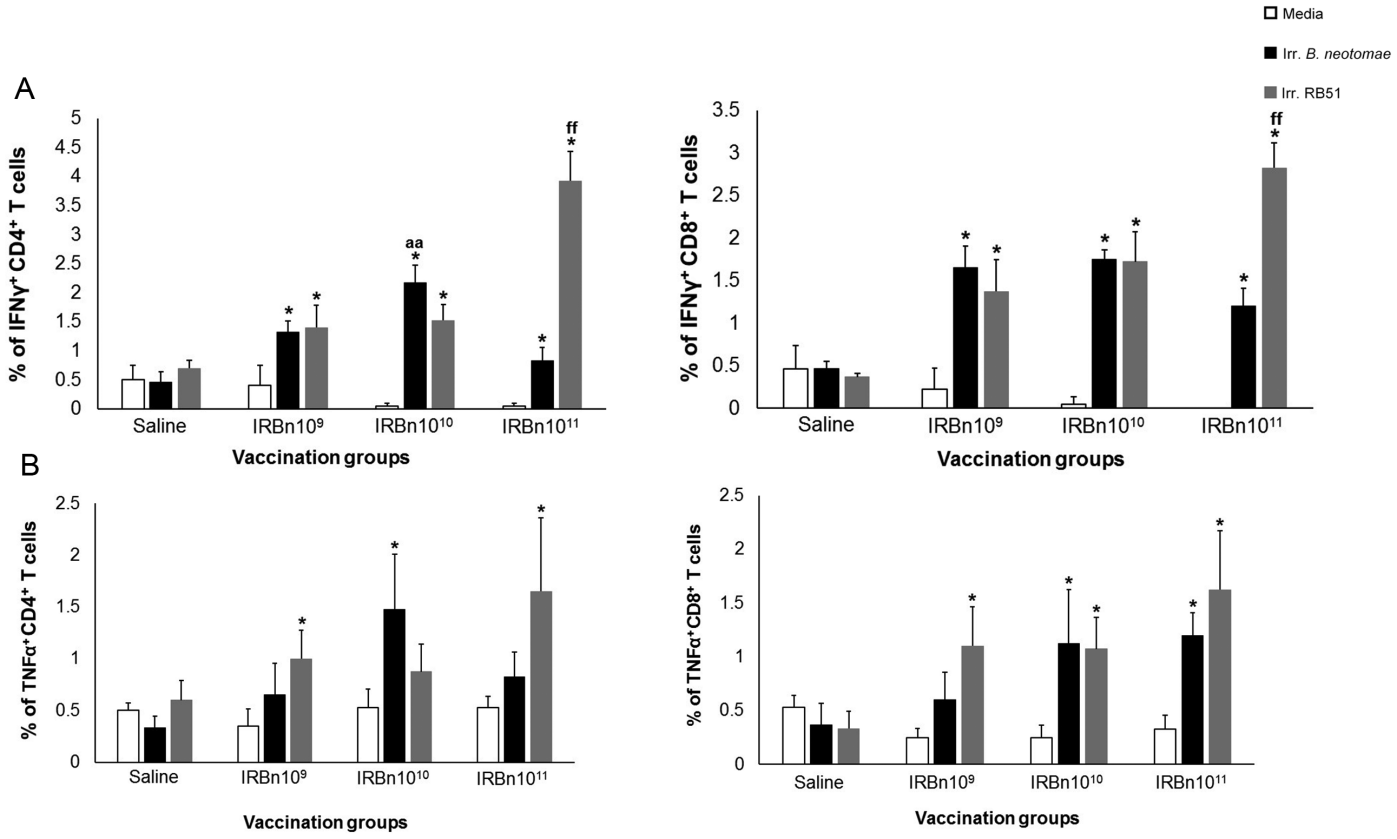
Flow cytometric analysis showing the percentage of interferon-γ secreting (A), and tumor necrosis factor-α secreting (B) CD4^+^ and CD8^+^ T cells in the spleens of BALB/c mice immunized with 10^9^ CFU-equivalent or 10^10^ CFU-equivalent or 10^11^ CFU-equivalent of gamma-irradiated *B. neotomae*, or inoculated with saline following in vitro stimulation with media (unstimulated), gamma-irradiated *B. neotomae* and gamma-irradiated RB51. *Significantly different from the corresponding unstimulated control within a vaccination group (*P*<0.05). ^aa^ Significantly different from the corresponding vaccination groups with irradiated *B. neotomae* stimulation (*P*<0.05). ^ff^ Significantly different from the corresponding vaccination groups with irradiated RB51 stimulation (*P*<0.05).

Upon in vitro stimulation of splenocytes with irradiated *B. neotomae* and irradiated RB51, significantly higher proportions of IFN-γ secreting CD4^+^ and CD8^+^ T cells were detected in all vaccinated groups (Fig. 6A). Stimulation of splenocytes with irradiated RB51 resulted in induction of significantly increased proportions of CD4^+^ and CD8^+^ T cells secreting IFN-γ in mice immunized with the 10^11^ CFU equiv. dose than the other groups (Fig. 6A). Significantly increased proportion of TNF-α secreting CD4^+^ and CD8^+^ T cells were detected upon stimulation of splenocytes of vaccinated mice with specific antigens (Fig. 6B).

### 6. Protective efficacy against intra-peritoneal and intra-nasal challenge with virulent *B. abortus* 2308 following oral homologous prime-boost immunization with different vaccine doses of gamma-irradiated *B. neotomae*

Upon i.p. challenge with *B. abortus* 2308, significantly reduced bacterial loads were detected in spleens and livers of all vaccinated mice (Table. 3); mice immunized with the 10^11^ CFU-equiv. dose showed the highest resistance with an average of 2.61 log and 2.49 log reduction in spleens and livers, respectively. Although the lung bacterial burden in all vaccinated groups was lower than that of the saline inoculated control group, the difference was not statistically significant (Table. 3); again, mice immunized with the 10^11^ CFU-equiv. dose showed the highest resistance in lungs with a 1.47 log difference in bacterial load compared to those of the saline control group.

**Table 3:** Protection against intra-peritoneal challenge with virulent *B. abortus* 2308 following oral homologous prime-boost immunization of mice with multiple vaccine doses of gamma-irradiated *B. neotomae*.

	Vaccine (gamma-irradiated)	Bacterial load in log_10_ CFU (mean ± SD)	Units of protection^a^
Log_10_ CFU in spleen	None (saline)	6.53±0.03	-
	*B. neotomae* 10^9^	5.27±0.53*	1.26
	*B. neotomae* 10^10^	5.18±0.50*	1.35
	*B. neotomae* 10^11^	3.92±1.29*	2.61
Log_10_ CFU in liver	None (saline)	4.48±0.19	-
	*B. neotomae* 10^9^	3.47±0.27*	1.01
	*B. neotomae* 10^10^	3.69±0.61*	0.79
	*B. neotomae*10^11^	1.99±1.64*	2.49
Log_10_ CFU in lung	None (saline)	3.61±0.88	-
	*B. neotomae* 10^9^	3.47±0.32**	0.14
	*B. neotomae* 10^10^	3.16±0.28**	0.45
	*B. neotomae*10^11^	2.14±1.15**	1.47

a Units of protection were calculated by subtracting the mean log_10_ CFU for a vaccinated group from the mean log_10_ CFU of the corresponding saline control group.

*Significantly different from the corresponding saline group (*P*<0.05).

**Not significantly different from the corresponding saline group (*P*>0.05).

Following intranasal challenge with *B. abortus* 2308, the bacterial CFUs in lungs and spleens of mice immunized with the 10^11^ CFU-equiv. dose were significantly lower than those of mice inoculated with saline (Table. 4). Reduced bacterial burden was also detected in the livers of mice immunized with the 10^11^ CFU-equiv. dose, though the reduction was not statistically significant from the saline group (Table. 4).

**Table 4:** Protection against intra-nasal challenge with virulent *B. abortus* 2308 following oral homologous prime-boost immunization of mice with multiple vaccine doses of gamma-irradiated *B. neotomae*.

	Vaccine (gamma-irradiated)	Bacterial load in log_10_ CFU (mean ± SD)	Units of protection^a^
Log_10_ CFU in spleen	None (saline)	5.10±0.61	-
	*B. neotomae* 10^9^	5.23±0.74	-
	*B. neotomae* 10^10^	5.34±0.39	-
	*B. neotomae* 10^11^	3.89±0.31*	1.21
Log_10_ CFU in liver	None (saline)	3.33±0.65	-
	*B. neotomae* 10^9^	3.36±0.48	-
	*B. neotomae* 10^10^	3.25±0.63**	0.08
	*B. neotomae*10^11^	2.12±1.12**	1.21
Log_10_ CFU in lung	None (saline)	5.30±0.12	-
	*B. neotomae* 10^9^	5.40±0.21	-
	*B. neotomae* 10^10^	4.67±0.94**	0.63
	*B. neotomae*10^11^	4.08±0.91*	1.22

a Units of protection were calculated by subtracting the mean log_10_ CFU for a vaccinated group from the mean log_10_ CFU of the corresponding saline control group.

*Significantly different from the corresponding saline group (*P*<0.05).

**Not significantly different from the corresponding saline group (*P*>0.05).

## Discussion

Vaccination by oral route is a desirable method to induce acquired immunity against infectious diseases in humans as well as animals. In most human brucellosis cases, the infection is acquired through mucosal routes. However, human brucellosis is a systemic disease, where the bacteria penetrate the epithelial barrier, spread to mononuclear phagocytic system, and affect different organ systems [19]. Therefore, an effective oral brucellosis vaccine must induce systemic immunity. Several research groups previously used live attenuated *Brucella* strains as oral vaccines [18] [20] and [21]. The inherent safety risks associated with bacterial replication may preclude the use of attenuated *Brucella* strains as live vaccines for human brucellosis. Our previous research demonstrated that gamma-irradiated *B. abortus* strain RB51 and *B. neotomae* cannot replicate, but can induce protective antibody and CMI responses when used as vaccines to immunize mice by intraperitoneal route [11] and [12]. In this study, we asked if these vaccines can be administered by oral route to immunize mice against a challenge infection with virulent *B. abortus*. Our empirical selection to give multiple doses of the vaccine was driven by the currently recommended oral vaccination regimen for *Salmonella typhi*, the only oral bacterial vaccine in human use at present, which consists of 4 doses at 1-day intervals [22]. We first examined the effect of prime-boost strategy on induction of immune responses and protection. Contrary to our initial hypothesis, priming with *B. neotomae* and boosting with *B. abortus* RB51, or vice versa, did not significantly change the type of antibody and CMI responses. The only notable difference was that the mice primed with *B. abortus* RB51 and boosted with *B. neotomae* did not develop significant levels of IgG1 antibodies specific to smooth LPS even at 2 weeks p.i. (Fig. 1A). It is interesting that priming with *B. neotomae* and boosting with *B. abortus* RB51 did not dampen the overall antibody response to smooth LPS (Fig. 1A). In fact, mice subjected to this vaccination regimen had significantly higher levels of smooth LPS-specific IgG2a and IgG2b antibodies at 1 week p.i., but the difference disappeared by 2 weeks p.i. (Fig. 1A). Majority of the surface proteins of *Brucella* spp. are highly conserved [23]. Therefore, it is not surprising that all tested vaccination regimens induced similar levels of RB51-specific antibodies in serum and intestinal secretions (Fig. 1B and 2B).

Our detection of smooth LPS- and RB51-specific antibodies in the intestinal secretions of the vaccinated mice is suggestive of induction of a mucosal immune response. However, the role of mucosal immunity in protection against brucellosis remains to be determined. A recent study demonstrated that mice orally vaccinated with live attenuated strains *B. abortus* RB51 or *B. melitensis* ΔznuA developed both systemic and mucosal Th1 as well as Th17 responses, but the Th17 responses played a role only in the *B. abortus* RB51-induced protection [21]. The important role of Th1 responses in protection against brucellosis is well-documented in the literature [4]. All the vaccines tested in our study induced antigen-specific CD4^+^ and CD8^+^ T cells that secrete IFN-γ or TNF-α in spleens, suggesting the generation of a systemic Th1 response.

In mouse brucellosis models, reduced bacterial load in spleens, livers or both, of the vaccinated mice compared to those of the unvaccinated animals is the generally used criterion for determining the vaccine-induced protective responses [24] [25] and [26]. Based on this standard, only mice vaccinated with *B. neotomae* and IRBn/RB51, but not RB51, developed significant protection against intraperitoneal challenge with *B. abortus* 2308 (Table 2). This was corroborated by the reduced bacterial burden in lungs also, though the reduction was not significantly different from the unvaccinated mice. This observation suggests that oral vaccines that elicit antibodies to smooth LPS are effective in affording protection in mouse brucellosis models. Since the protection induced by the IRBn/RB51 vaccination regimen was not different from that of the IRBn vaccine, we then examined the effect of reduced dose of *B. neotomae* vaccine on induction of protection. All tested vaccine doses induced significant protection against the intraperitoneal challenge. However, only the highest dose provided significant protection against the intranasal challenge. Why the lower doses of vaccine could not provide protection against the intranasal challenge could not be answered based on our immune response analyses. There was a trend of reduced serum and mucosal antibody levels to smooth LPS with reduced vaccine dose, which could have contributed to this difference. A thorough analysis of T cell responses in lungs following immunization and challenge may reveal important differences between the different vaccination doses. We did attempt to measure the cellular immunity in the mediastinal lymph nodes of orally vaccinated mice prior to challenge. Unlike in splenocyte cultures, no significant level of IFN-γ was detected in the mediastinal lymph node cell cultures upon stimulation with specific *Brucella* antigens. It is possible that analysis of T cells homing to lungs and their responses following challenge infection is needed to determine the protective correlates in lungs. Additionally, determining the effect of different vaccine doses on the persistence of the vaccine bacteria in lymphoid organs may also yield information relevant to understanding the magnitude of immune responses induced at different mucosal sites. Our preliminary study indicates that following oral inoculation of mice with 10^11^ CFU-equivalent of gamma-irradiated *B. neotomae*, the bacterial DNA could be detected by quantitative real-time PCR in mesenteric lymph nodes and in spleens by day 1 and persisted there for at least three days (Figure S1).

**Table 2:** Protection against intra-peritoneal challenge with virulent *B. abortus* 2308 following oral prime-boost immunization of mice with gamma-irradiated *B. neotomae* and/or gamma-irradiated *B. abortus* RB51.

	Vaccine (gamma-irradiated)	Bacterial load in log_10_ CFU (mean ± SD)	Units of protection^a^
Log_10_ CFU in spleen	None (saline)	4.66±0.77	-
	RB51	4.03±0.93**	0.63
	*B. neotomae*	2.55±0.21*	2.11
	*B. neotomae*/RB51	2.97±0.22*	1.69
Log_10_ CFU in liver	None (saline)	3.61±0.11	-
	RB51	2.82±0.32*	0.79
	*B. neotomae*	2.53±0.21*	1.08
	*B. neotomae*/RB51	2.62±0.25*	0.99
Log_10_ CFU in lung	None (saline)	2.59±1.18	-
	RB51	2.71±0.53	-
	*B. neotomae*	1.85±0.46**	0.74
	*B. neotomae*/RB51	1.70±0.28**	0.89

a Units of protection were calculated by subtracting the mean log_10_ CFU for a vaccinated group from the mean log_10_ CFU of the corresponding saline control group.

*Significantly different from the corresponding saline group (*P*<0.05).

**Not significantly different from the corresponding saline group (*P*>0.05).

In conclusion, these studies demonstrate the feasibility of using gamma-irradiated *B. neotomae* as oral vaccine to induce protection against both parenteral and mucosal challenge with virulent *Brucella* spp. This type of vaccine is a safer alternative to live vaccines for human brucellosis. Future studies should focus on formulating the vaccine to bypass the acidic conditions of human stomach, assessing the shelf-life of the vaccines and determining the duration of the vaccine-induced protective response.

## Supporting Information

Figure S1 **Persistence of gamma-irradiated** ***B. neotomae*** **in mouse spleens and mesenteric lymph nodes as detected by real-time quantitative PCR.** A group of 8 female BALB/c mice were orally administered with 1×10^11^ CFU-equivalent of gamma-irradiated *B. neotomae*. On days 1 and 3 post-vaccination, 4 mice from the group were euthanized and their spleens and mesenteric lymph nodes were collected aseptically. The organs were homogenized in PBS and DNA from the homogenates were extracted using a commercial kit (DNeasy Blood and Tissue Kit, Qiagen Inc.). Quantification of *B. neotomae* DNA in the samples was accomplished using real-time PCR as previously described (Moustafa et al. Vaccine 2011; 29(4): 784–794).
(TIF)
