# Supplementary figures and images for: Oral Immunization of Mice with Gamma-Irradiated Brucella neotomae Induces Protection against Intraperitoneal and Intranasal Challenge with Virulent B. abortus 2308
Source: PLoS One. 2014 Sep 16;9(9):e107180. doi: 10.1371/journal.pone.0107180 (PMC4166413; doi:10.1371/journal.pone.0107180)

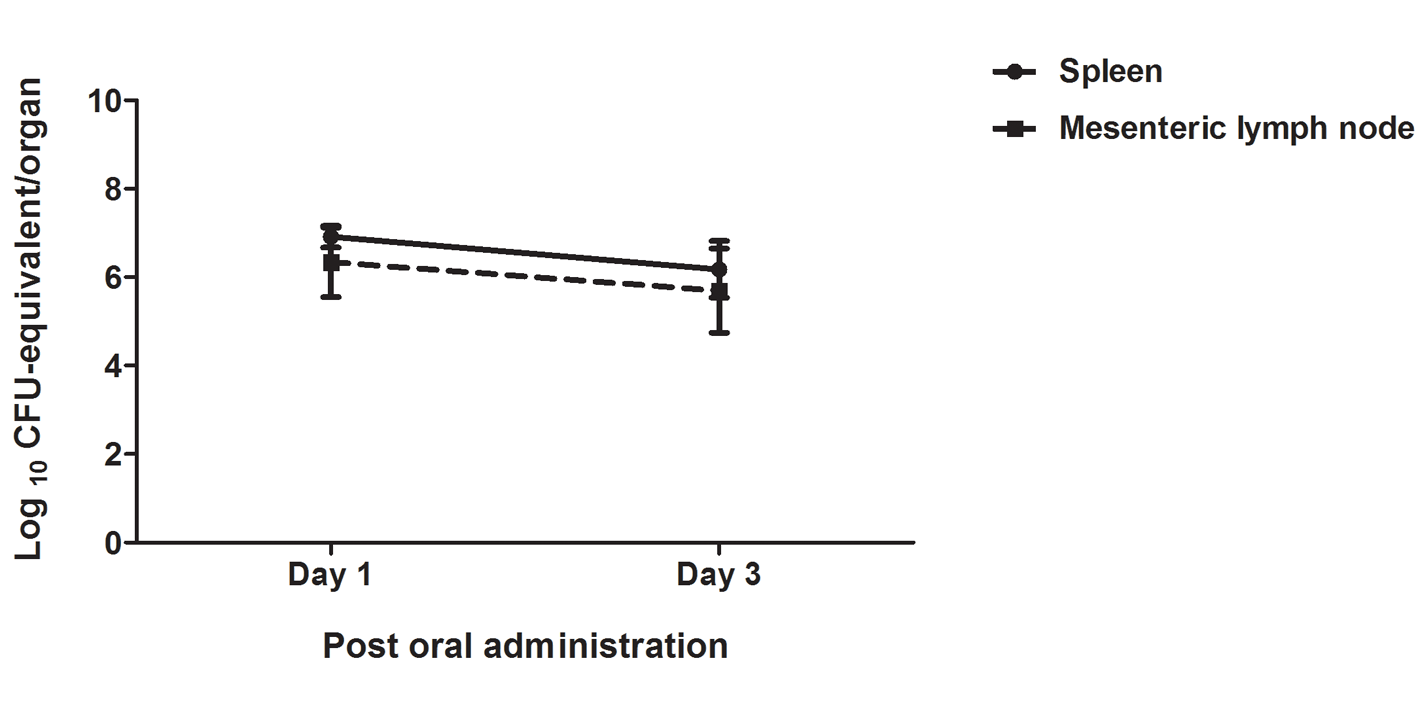

Supplement: Figure S1 — Persistence of gamma-irradiated B. neotomae in mouse spleens and mesenteric lymph nodes as detected by real-time quantitative PCR. A group of 8 female BALB/c mice were orally administered with 1×10^11^ CFU-equivalent of gamma-irradiated B. neotomae. On days 1 and 3 post-vaccination, 4 mice from the group were euthanized and their spleens and mesenteric lymph nodes were collected aseptically. The organs were homogenized in PBS and DNA from the homogenates were extracted using a commercial kit (DNeasy Blood and Tissue Kit, Qiagen Inc.). Quantification of B. neotomae DNA in the samples was accomplished using real-time PCR as previously described (Moustafa et al. Vaccine 2011; 29(4): 784–794). (TIF) [file pone.0107180.s001.tif]
